# Ginsenoside C-K inhibits Aβ oligomer-induced Alzheimer's disease pathology progression by regulating microglia-neuron interactions

**DOI:** 10.1016/j.ibneur.2025.05.007

**Published:** 2025-05-21

**Authors:** Chenghu Xie, Cunxin Zhang, Kefeng Zhang, Shanshan Zhang

**Affiliations:** a Department of Spine Surgery, Jining NO.1 People's Hospital Affiliated to Shandong First Medical University & Shandong Academy of Medical Sciences, China; b Shandong Provincial Key Medical and Health Laboratory of Neuroinjury and Repair, China; c Department of Neurology, Jining NO.1 People's Hospital Affiliated to Shandong First Medical University & Shandong Academy of Medical Sciences, China

**Keywords:** Alzheimer's disease, Ginsenoside C-K, Amyloid-β oligomer, Microglia-neuron crosstalk, NF-κB signaling pathway, Neuroinflammation

## Abstract

**Background:**

Alzheimer’s disease is a progressive neurodegenerative disorder. Current therapeutic agents primarily focus on symptom alleviation and fail to effectively halt disease progression. Therefore, there is a need to develop novel therapeutic strategies, particularly those involving natural active compounds with multi-target actions.

**Objective:**

To investigate the intervention effects and multi-target regulatory mechanisms of Ginsenoside C-K (GCK) on β-amyloid (Aβ) oligomer-induced Alzheimer's disease (AD) pathological progression.

**Methods:**

BV2 microglia and HT22 neurons were used as in vitro models. Cell viability was measured via CCK-8 assay, cell migration ability assessed by scratch assay, and apoptosis rate analyzed using Annexin V/PI dual staining. A conditioned medium (CM) strategy was employed to validate microglia-neuron interactions. Western blot was performed to detect key NF-κB signaling pathway proteins (p-IκBα, p-p65) and inflammatory cytokines (TNF-α, IL-1β).

**Results:**

GCK pretreatment significantly ameliorated Aβ₁₋₄₂ oligomer-induced BV2 cell dysfunction (viability recovery rate >80 %, p < 0.01), suppressed pro-inflammatory cytokine secretion (TNF-α reduced by 62.3 %, IL-1β by 57.8 %), and inhibited NF-κB pathway activation (p-IκBα/p-p65 expression downregulated >50 %). In HT22 neurons, GCK directly counteracted Aβ toxicity (apoptosis rate decreased from 38.7 % to 15.2 %) and exerted indirect neuroprotection by modulating microglia-derived conditioned medium (CM2 group showed a 2.1-fold increase in neuronal viability compared to CM1).

**Conclusion:**

GCK mitigates AD pathology through dual mechanisms-direct inhibition of Aβ neurotoxicity and indirect regulation of microglial homeostasis-with NF-κB signaling suppression as a core mechanism. This study provides new experimental evidence for natural product-based multi-target AD therapies, though further animal studies are required to validate its in vivo efficacy and safety.

## Highlights

• GCK inhibits Aβ-induced neurotoxicity and inflammation.
• GCK regulates microglia-neuron interactions via NF-κB pathway.
• GCK reduces apoptosis and enhances neuronal viability.
• GCK shows potential as a multi-target AD therapeutic agent.

## 1 Introduction

Alzheimer's Disease (AD), as a progressive neurodegenerative disorder, has become a global public health challenge (Anon, 2024). According to World Health Organization statistics, there are currently approximately 50 million dementia patients worldwide, with AD accounting for 60–70 % of cases. With the accelerating global population aging, the number of dementia patients is projected to reach 82 million by 2030 and exceed 150 million by 2050. In China, the number of AD patients has already reached approximately 10 million, growing by 300,000 annually, while the disease burden continues to escalate, urgently requiring effective treatment solutions (Ji et al., 2024). Currently, FDA-approved AD medications primarily fall into three categories: acetylcholinesterase inhibitors (Conti et al., 2023) (such as donepezil, rivastigmine, and galantamine), NMDA receptor antagonists (Ippoliti et al., 2023) (such as memantine), and novel anti-amyloid monoclonal antibodies (Haddad et al., 2022) (such as aducanumab). However, these existing therapies mainly focus on symptomatic improvement and cannot fundamentally halt disease progression. Consequently, developing novel therapeutic agents-particularly natural active ingredients with multi-target synergistic effects-has emerged as a critical research priority.

The pathogenesis of Alzheimer's disease (AD) is characterized by abnormal metabolism and deposition of amyloid-beta (Aβ). Under normal physiological conditions, Aβ is generated through sequential cleavage of amyloid precursor protein (APP) by β-secretase and γ-secretase, and subsequently cleared from brain tissue via multiple pathways (Ahn and Park, 2025). In pathological states, increased Aβ production or reduced clearance leads to its abnormal aggregation in extracellular spaces. These Aβ aggregates gradually form oligomers and fibrils with β-sheet structures, ultimately depositing as insoluble amyloid plaques (Wee et al., 2023). Plaque formation triggers a cascade of inflammatory responses, in which microglia play a pivotal role. As immune cells of the central nervous system, microglia recognize and phagocytose Aβ, exerting protective effects in early stages. However, persistent Aβ stimulation during disease progression causes microglial overactivation, prompting excessive secretion of pro-inflammatory factors such as interleukin-1β (IL-1β), tumor necrosis factor-α (TNF-α), and interferon-γ (IFN-γ) (Xu et al., 2022). These inflammatory mediators exacerbate neuroinflammation, creating a self-perpetuating cycle. Hyperactivated microglia also generate reactive oxygen species (ROS) and nitric oxide (NO), substances with direct cytotoxic effects on neurons (Qian et al., 2024). Concurrently, Aβ directly impairs synaptic function, disrupts cell membrane integrity, and activates apoptotic pathways in neurons. Sustained inflammation induces neuronal mitochondrial dysfunction, calcium homeostasis imbalance, and increased oxidative stress, ultimately leading to synaptic loss and neuronal death (Reinders et al., 2025; Wu et al., 2024). This pathogenic cascade-initiated by Aβ deposition, amplified through microglia-mediated neuroinflammation, and culminating in neuronal dysfunction and death-constitutes the core mechanism of AD pathogenesis. As the disease progresses, these pathological processes mutually reinforce one another, forming an intractable vicious cycle.

Ginsenoside C-K (GCK), the primary active metabolite of ginsenoside Rb1 in vivo, exhibits significant anti-inflammatory, antioxidant, and cytoprotective effects. Studies demonstrate that GCK effectively modulates the activation state of macrophages by suppressing the NF-κB signaling pathway, reducing the ratio of M1 to M2 macrophages, thereby significantly inhibiting inflammatory responses in murine collagen-induced arthritis (CIA) and slowing disease progression (Wang et al., 2019a, Wang et al., 2019b). Zhang et al. revealed that the ginsenoside metabolite C-K suppresses B cell activation and ameliorates CIA by promoting IgD-type B cell receptor (IgD-BCR) endocytosis via a β-arrestin1-AP2-dependent mechanism (Zhang et al., 2019). Additionally, Liu et al. discovered that C-K inhibits abnormal activation and differentiation of T lymphocytes by regulating expression levels of T cell receptor (TCR), CD28, CTLA-4, and PD-1, consequently attenuating CIA progression (Liu et al., 2014). Given GCK's regulatory effects on immune cells, we hypothesize that GCK may mitigate neuroinflammation and ameliorate Alzheimer's disease (AD) progression by modulating the polarization state of microglia.

In terms of cytoprotection, GCK enhances cellular antioxidant capacity by activating the Nrf2/HO-1 pathway, mitigating oxidative stress damage and improving neuronal synaptic dysfunction (Li et al., 2023). Through activation of the PI3K pathway, it increases the activity of protein kinase B (Akt) and endothelial nitric oxide synthase (eNOS), while suppressing calcium ion-induced mitochondrial swelling and inhibiting myocardial ischemia-reperfusion injury (Tsutsumi et al., 2011). This multi-target mechanism positions GCK as a potential therapeutic agent against β-amyloid-induced neurotoxicity, reducing Aβ deposition and hyperphosphorylation of Tau protein. By simultaneously targeting microglia-mediated inflammatory responses and neuronal survival mechanisms, GCK demonstrates unique therapeutic advantages. These findings provide novel insights for Alzheimer's disease treatment, suggesting that GCK may serve as an effective therapeutic agent to improve cognitive function through dual mechanisms of suppressing neuroinflammation and protecting neurons.

In vitro studies have demonstrated that GCK significantly inhibits the activation of the NF-κB signaling pathway, alleviates inflammatory responses in microglial cells, and reduces the release of pro-inflammatory mediators such as TNF-α and IL-1β, while simultaneously promoting the expression of anti-inflammatory substances. This effectively improves the inflammatory microenvironment in AD. Furthermore, GCK has shown direct inhibitory effects on Aβ42 oligomer-mediated neurotoxicity, mitigating neuronal damage caused by Aβ deposition through multi-target mechanisms. These findings provide a critical theoretical foundation for GCK as a potential therapeutic agent for AD, while also establishing new research directions for developing natural active components with multi-target synergistic effects.

## 2 Materials and methods

### 2.1 Cell culture

The HT22 mouse hippocampal neuron cell line and BV2 microglial cell line were purchased from Procell Life Science & Technology Co., Ltd. (Wuhan, China; official website: www.procell.com.cn). The cells were routinely cultured in their respective dedicated media: HT22 cell-specific medium (containing 10 % fetal bovine serum and 1 % penicillin-streptomycin dual antibiotic solution) and BV2 cell-specific medium (containing 10 % fetal bovine serum and 1 % penicillin-streptomycin dual antibiotic solution). When cell confluence reached 90 %, the cells were digested with 0.25 % trypsin (containing 0.02 % EDTA) and subcultured at a 1:3 ratio into 6-well plates (2 × 10^5 cells/well). After subculturing, the cells were placed in a 37 °C, 5 % CO₂ constant-temperature incubator for synchronized culture over 24 h. Subsequent experimental procedures, including grouping according to the experimental design and drug administration interventions, were conducted prior to downstream experimental analyses.

### 2.2 Cell viability assay

The Cell Counting Kit-8 (Dojindo, Japan) was used to quantitatively assess cell proliferation viability. The specific procedure was as follows: First, the original culture medium was aspirated and discarded, followed by two washes with PBS. Serum-free medium containing 10 % CCK-8 reagent (1:10 vol ratio) was then added to each well, and the plates were incubated at 37 °C in a 5 % CO₂ atmosphere under light-protected conditions. Optical density (OD) values were measured at 1, 2, 3, and 4 h post-incubation using a multi-mode microplate reader (BioTek Instruments, USA) with dual wavelengths (detection wavelength 450 nm/reference wavelength 630 nm). Cell viability was calculated using the formula: Cell viability (%) = [(OD experimental group - OD blank group)/(OD control group - OD blank group)] × 100 %.

### 2.3 Cell scratch assay

Cell migration ability was assessed using a standardized scratch wound healing assay. When the cell monolayer reached 95 % confluency, uniform-width scratches (500 ± 20 μm) were created vertically along the culture dish bottom under sterile conditions using a 200 μL sterile pipette tip (Catalog No. T-200-Y, Axygen). To ensure experimental accuracy, pre-marked reference points on the culture dish bottom were used to capture baseline images (t = 0 h) under an Olympus IX73 inverted phase-contrast microscope (10× objective). Cells were then returned to a 37 °C, 5 % CO₂ incubator for continued culture. After 24 h, images were reacquired from the same pre-marked regions (t = 24 h). Scratch area changes were measured using Image J software (NIH, v1.53), and cell migration rates were calculated accordingly. The calculation formula for cell migration rate is as follows:

Cell migration rate (%) =[(A₀ - Aₜ) / A₀] × 100%

A₀: Initial scratch area (t = 0 h), At: Remaining scratch area (t = 24 h).

### 2.4 Flow cytometry analysis

The regulatory effects of ginsenosides on cell apoptosis were quantitatively assessed using an Annexin V-FITC/PI dual-staining apoptosis detection kit (Bestbio, Nanjing, China). Post-treatment cells from each group were digested, collected, and resuspended in buffer to a final concentration of 1 × 10⁶ cells/mL. A 100 μL aliquot of cell suspension was sequentially supplemented with 5 μL Annexin V-FITC and 5 μL PI, followed by precise incubation at 37 °C in the dark for 15 min. Concurrent controls included an Annexin V-FITC single-stained group, PI single-stained group, and unstained negative control group for instrument compensation. Detection was performed using a CytoFLEX LX flow cytometer (Beckman Coulter, USA), with FITC signals captured through a 525/40 nm optical filter and PI signals collected via a 620/30 nm filter. A minimum of 100,000 valid cell events were acquired per sample. Data were analyzed using CytExpert 2.4 software, with early apoptotic (Annexin V⁺/PI⁻) and late apoptotic (Annexin V⁺/PI⁺) cell proportions determined via four-quadrant analysis. Experiments were repeated three times, with data expressed as mean ± standard deviation.

### 2.5 Enzyme-linked immunosorbent assay (ELISA)

The levels of inflammatory factors were quantitatively measured using a sandwich ELISA method (SPbio, Wuhan, China). The procedure was performed as follows: After equilibrating a 96-well plate pre-coated with specific capture antibodies at room temperature for 30 min, 100 μL/well of standards (concentration gradient: 0–1000 pg/mL, 8 concentration points) or pretreated test samples was added. Sample pretreatment involved low-speed centrifugation (3000 rpm, 10 min, 4 °C) to remove impurities, followed by a 1:2 dilution. The plate was incubated at 37 °C for 2 h and then washed five times with phosphate-buffered saline containing 0.05 % Tween-20 (PBST, pH 7.4) (300 μL/well). Next, 100 μL of biotinylated detection antibody (1:100 dilution) was added, and the plate was incubated at 37 °C for 60 min. After washing, 100 μL of horseradish peroxidase (HRP)-conjugated streptavidin (1:200 dilution) was added, followed by a 30-min incubation at 37 °C in the dark. After the final wash, 90 μL of TMB chromogenic substrate was added, and the plate was incubated in the dark at room temperature (25 ± 2 °C) for 15 ± 1 min. Following the addition of 50 μL of stop solution (2 M H₂SO₄), the optical density (OD) was immediately measured at 450 nm (reference wavelength 630 nm) using a Bio Tek Synergy HTX multi-mode microplate reader (Bio Tek Instruments, USA).

### 2.6 RNA extraction and quantitative reverse transcription PCR (qRT-PCR)

Total RNA was extracted using the Fast Pure Cell/Tissue Total RNA Isolation Kit V2 (Vazyme Biotech, Nanjing, China). The procedure was as follows: Cells in the logarithmic growth phase were collected and lysed with 1 mL TRIzol reagent. Total RNA was obtained through chloroform extraction, isopropanol precipitation, and 75 % ethanol washing. RNA purity (A260/A280 ratio 1.8–2.0) and concentration were measured using a Nanodrop 2000 spectrophotometer (Thermo Fisher Scientific). Reverse transcription was performed with the HiScript III RT SuperMix for qPCR (+gDNA-wiper) kit (Vazyme Biotech, Nanjing, China). For each reaction, 1 μg of total RNA was treated with gDNA wiper for 15 min to remove genomic DNA contamination. cDNA synthesis was conducted in a 20 μL optimized reaction system: 42 °C for 15 min, followed by 85 °C for 5 s to inactivate the reverse transcriptase.

Quantitative real-time PCR (qPCR) was performed using Taq Pro Universal SYBR qPCR Master Mix (Vazyme Biotech, Nanjing, China) on a Bio-Rad CFX Maestro 2.2 real-time PCR system (Bio-Rad, USA). The 20 μL PCR reaction mixture contained 10 μL 2 × SYBR Green Premix, 0.4 μL forward primer (10 μM), 0.4 μL reverse primer (10 μM), 2 μL template cDNA (1:5 dilution), and 7.2 μL nuclease-free water. The amplification protocol included: 95 °C for 3 min; 40 cycles of 95 °C for 10 s and 60 °C for 30 s; followed by melt curve analysis (65–95 °C, 0.5 °C increments every 5 s). β-actin was used as the reference gene, and relative target gene expression was calculated using the 2^(-ΔΔCt) method. All primers, synthesized by Sangon Biotech (Shanghai, China) with HPLC purification, are listed in Table 1.

**Table 1:** Sequences of primers used in the study.

	Direction	Sequences
IL−1β	Forward(5’−3’)	GCCACCTTTTGACAGTGATG
	Reverse(5’−3’)	GTGCTGCTGCGAGATTTGAA
TNF-ɑ	Forward(5’−3’)	GATCGGTCCCAACAAGGAGG
	Reverse(5’−3’)	GCTTGGTGGTTTGCTACGAC
β-actin	Forward(5’−3’)	CGATATCGCTGCGCTGGTC
	Reverse(5’−3’)	AGGTGTGGTGCCAGATCTTC

### 2.7 Western blot

The target protein expression was quantitatively analyzed using Western blot. The detailed procedure is as follows: Total proteins were extracted using modified RIPA lysis buffer (Beyotime, Nanjing, China) with a ratio of RIPA:PMSF (1 mM):phosphatase inhibitor = 100:1:2 (v/v/v). After homogenization (60 Hz, 2 × 45 s), samples were centrifuged at 14,000 × g for 15 min at 4 °C to collect supernatants. Protein concentrations were determined by BCA method (Beyotime, Nanjing, China), and 30 μg of total protein was mixed with 5× SDS-PAGE loading buffer followed by denaturation at 100 °C for 5 min. Proteins were separated by 10 % separating gel electrophoresis (120 V, 90 min) and transferred to PVDF membranes (0.45 μm, Millipore) using a semi-dry transfer system (25 V, 30 min). After blocking with 5 % BSA-TBST (room temperature, 60 min), membranes were incubated with specific primary antibodies overnight at 4 °C, washed with TBST, then incubated with HRP-conjugated secondary antibodies (1:5000) at room temperature for 60 min. Images were captured using ECL Plus developer (Vazyme Biotech) on a Tanon 5200 system, with grayscale analysis performed using ImageJ software using GAPDH as internal reference. The following antibodies were used: NF-κB antibody (Cat. No.: AF5006), phosphorylated NF-κB antibody (Cat. No.: AF2006), beta actin antibody (Cat. No.: AF7018) (all from Affinity Biosciences, China); IκBα antibody (Cat. No.: ab178846), phosphorylated IκBα antibody (Cat. No.: ab133462) (all from Abcam, Cambridge, UK).

### 2.8 Statistical analysis

The experimental data analysis was performed using SPSS 27.0 (IBM, USA) for data processing and GraphPad Prism 6.0 (GraphPad Software, USA) for statistical visualization. Normality of the data was assessed through Shapiro-Wilk tests combined with normal Q-Q plots. For quantitative data conforming to a normal distribution, one-way analysis of variance (ANOVA) was applied, followed by post hoc tests (Bonferroni or Tamhane's T2 methods). Measurement results are expressed as mean ± standard deviation (SD) from three independent replicate experiments, with a statistical significance threshold of P < 0.05.

## 3 Results

### 3.1 Determination of GCK's in vitro toxicity safety window and characterization of neuroprotective dose-effect in AD pathological models

#### 3.1.1 Cytotoxicity threshold determination and model construction

**Fig. 1:**
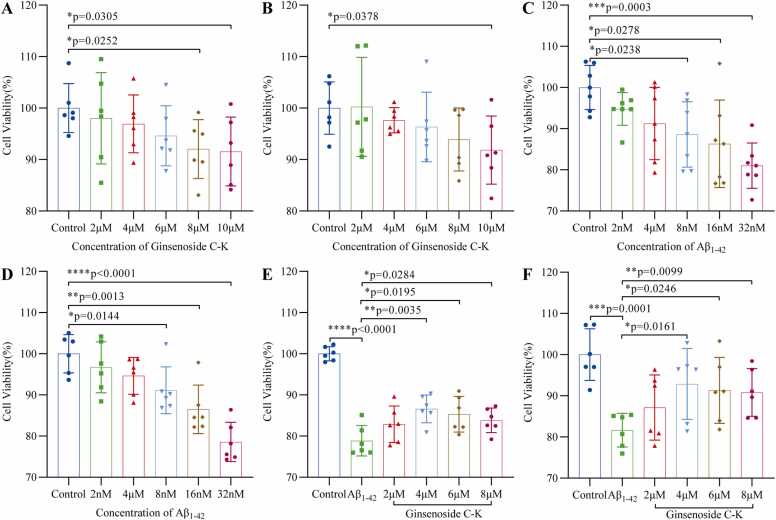
In vitro toxicity safety window determination of GCK and evaluation of its neuroprotective effects in Aβ pathology models. (A, B) Cytotoxicity analysis of GCK on HT22 and BV2 cells. Cell viability was measured using the CCK-8 assay after 24-h treatment with varying GCK concentrations (0, 2, 4, 6, 8, and 10 μM). Results showed that 8 μM GCK significantly inhibited HT22 cell viability (p < 0.05), while no significant toxicity was observed at 6 μM or lower concentrations. For BV2 cells, viability remained relatively high at 8 μM but significantly decreased at 10 μM (p < 0.05). (C, D) Construction of an Aβ1–42 oligomer-induced Alzheimer's disease in vitro pathology model. After 24-h treatment with Aβ1–42 oligomers (0, 2, 4, 8, 16, and 32 nM), cell viability in both HT22 and BV2 cells decreased significantly with increasing Aβ concentrations (p < 0.001). The 32 nM concentration was selected as optimal for modeling pathology, reducing survival rates to 81.03 ± 5.50 % in HT22 and 78.58 ± 4.37 % in BV2 cells. (E, F) Neuroprotective effects of GCK against Aβ1–42 oligomer-induced cellular damage. Pretreatment with GCK (2, 4, 6, and 8 μM) for 2 h followed by 24-h co-treatment with 32 nM Aβ1–42 oligomers revealed that 4 μM GCK provided the most significant protection (p < 0.05), restoring cell viability to levels comparable to the control group. Note: Data presented as mean ± SD (n = 6); *p < 0.05, **p < 0.01, ***p < 0.001.

To scientifically define the cellular safety window of Ginsenoside C-K (GCK), this study established a pharmacological foundation through dose-gradient cytotoxicity experiments. HT22 mouse hippocampal neurons and BV2 microglial cells were exposed to GCK solutions (0–10 μM) for 24 h, with cell viability assessed via CCK-8 assay. Results showed that GCK at 6 μM caused no significant cytotoxicity in HT22 cells (viability >95 %), but viability sharply decreased to 92.03 ± 5.21 % at 8 μM. BV2 cells maintained 93.90 ± 5.59 % viability at 8 μM, which significantly declined to 91.85 ± 6.04 % at 10 μM (Fig. 1A,B). Based on these findings, the safe working concentrations were established as ≤6 μM for HT22 cells and ≤8 μM for BV2 cells.

#### 3.1.2 AD pathological model establishment

To construct an in vitro AD model, Aβ_1–42_ oligomers (0–32 nM) were used for pathological simulation. After 24 h exposure, HT22 cells exhibited dose-dependent viability reduction, with 32 nM treatment showing 81.03 ± 5.50 % survival (vs control, p < 0.01), while BV2 cells demonstrated 78.58 ± 4.37 % survival (p < 0.001), both within the 60–80 % viability range suitable for modeling (Fig. 1C,D). This concentration was selected as the baseline injury dose for subsequent experiments. It is noteworthy that using HT22 cells as a model may not fully reflect the true pathological state of differentiated neurons in Alzheimer's disease.

#### 3.1.3 Neuroprotective concentration optimization

Within the safe dose framework (2–8 μM), GCK's antagonistic effects against Aβ toxicity were systematically evaluated. GCK pretreatment (2 h) followed by co-treatment with Aβ_1–42_ oligomers (32 nM, 24 h) revealed that 4 μM GCK exhibited optimal protective efficacy: HT22 cell viability increased to 86.61 ± 3.09 % (vs 78.87 ± 3.35 % in Aβ_1–42_ oligomers-only group), and BV2 cell viability recovered to 92.87 ± 7.85 % (vs 81.64 ± 3.72 % in Aβ_1–42_ oligomers-only group) (Fig. 1E,F). This concentration was therefore determined as the optimal intervention dose for subsequent mechanistic studies.

### 3.2 Neuroprotective mechanisms of GCK based on microglial homeostasis regulation

**Fig. 2:**
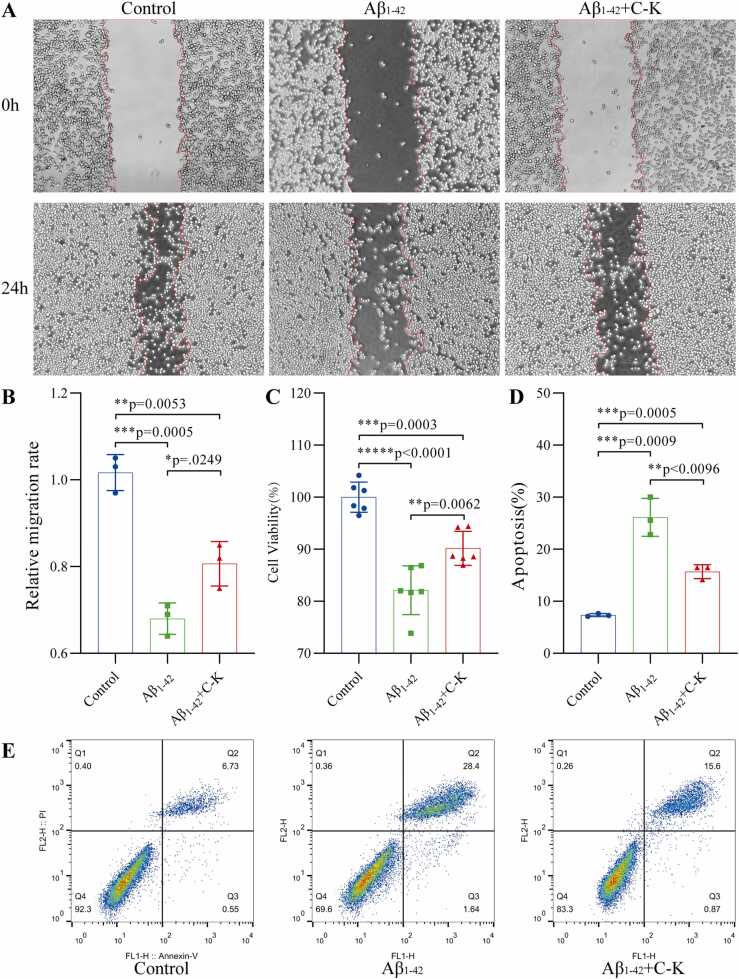
GCK exerts neuroprotective effects by regulating microglial homeostasis. (A) Scratch assay evaluating migration capacity: Control BV2 cells showed strong migration ability, with near-complete scratch closure after 24 h. Aβ_1–42_ oligomer (32 nM) treatment significantly inhibited scratch healing, while GCK (4 μM) pretreatment effectively restored BV2 cell migration. (B) Quantitative migration rate analysis: Compared to the control group, Aβ_1–42_ oligomer treatment significantly reduced migration rate (68.00 ± 2.94 %, p < 0.001). GCK pretreatment notably increased migration rate (80.67 ± 4.19 %, p < 0.05 vs Aβ group). (C) Cell viability assay: CCK-8 results demonstrated that Aβ_1–42_ oligomers significantly decreased BV2 cell viability (82.13 ± 4.28 %, p < 0.001 vs control), while GCK pretreatment restored viability to 90.18 ± 2.98 % (p < 0.01 vs Aβ group) (n = 6). (D, E) Apoptosis rate assessment: Annexin V/PI double-staining flow cytometry revealed Aβ1–42 oligomer treatment significantly increased apoptosis rate (26.14 ± 2.98 %, p < 0.001 vs control), whereas GCK pretreatment reduced apoptosis to 15.69 ± 1.10 % (p < 0.01 vs Aβ group). Note: Data expressed as mean ± SD (n = 3). C-K: Ginsenoside Compound K. *p < 0.05, **p < 0.01, ***p < 0.001.

The characteristic pathological alterations in Alzheimer’s disease (AD) originate from neuroinflammatory microenvironment disruption induced by dynamic imbalance of Aβ metabolism. This study found that 32 nM Aβ_1–42_ oligomer intervention significantly disrupted the functional homeostasis of BV2 microglial cells, manifesting as reduced viability (82.13 ± 4.28 % vs. control, p < 0.001), decreased migration capacity (68.00 ± 2.94 % in scratch assay, p < 0.001), and elevated apoptosis rate (26.14 ± 2.98 % via Annexin V/PI assay vs. control 7.35 ± 0.23 %, p < 0.001). Notably, 4 μM GCK pretreatment (2 h) exhibited multi-dimensional protective effects: restoring cell viability to 90.18 ± 2.98 % (p < 0.01 vs. Aβ group), enhancing migration capacity to 80.67 ± 4.19 % of the control level, and reducing apoptosis rate to 15.69 ± 1.10 % (p < 0.01). These findings suggest that GCK may reshape the immune microenvironment of the central nervous system by maintaining the dynamic equilibrium of microglial proliferation, migration, and apoptosis; however, it is crucial to validate these effects in differentiated neuronal models to better understand their relevance in AD pathology (Fig. 2).

### 3.3 GCK regulates Aβ1–42 oligomer-induced neuroinflammation by suppressing the NF-κB signaling pathway

**Fig. 3:**
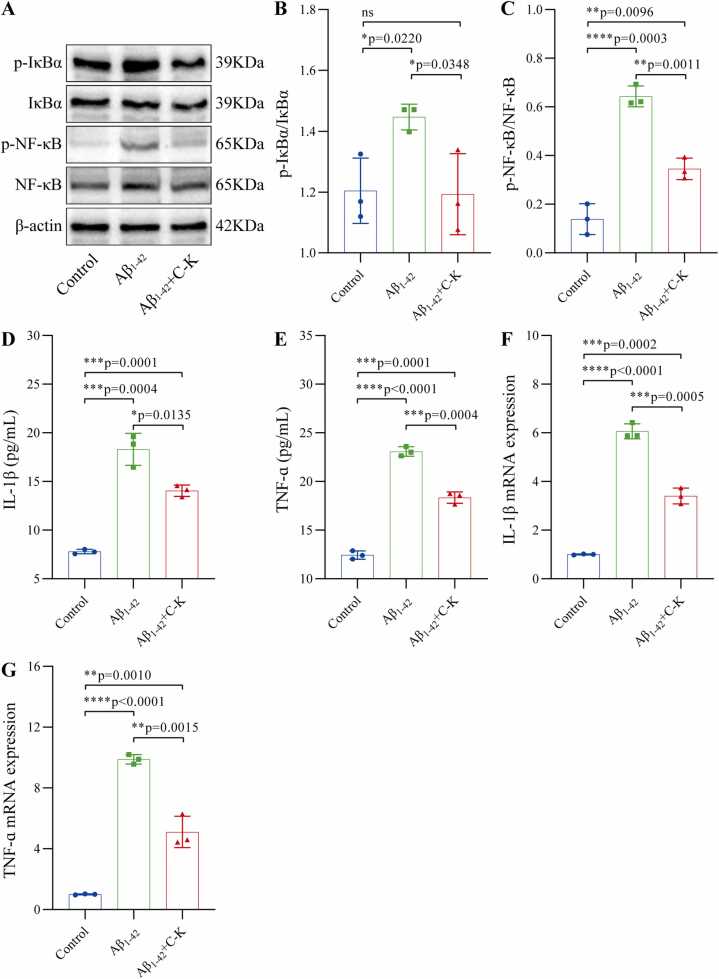
GCK alleviates Aβ1–42 oligomer-induced neuroinflammation by inhibiting the IκBα/NF-κB signaling pathway. (A-C) Western blot analysis showed that 32 nM Aβ1–42 oligomer stimulation significantly upregulated the expression of phosphorylated IκBα (p-IκBα, Ser32) and nuclear phosphorylated NF-κB p65 (p-NF-κB p65, Ser536) in BV2 cells, while 4 μM GCK pretreatment inhibited their phosphorylation and nuclear translocation. (D-F) ELISA assays demonstrated that IL-1β and TNF-α secretion levels increased to (18.31 ± 1.34) pg/mL and (23.08 ± 0.40) pg/mL (P < 0.01) after Aβ1–42 oligomer stimulation, while C-K pretreatment significantly reduced their secretion (*P < 0.01). These data indicate that GCK mitigates neuroinflammatory responses by blocking IκBα phosphorylation and NF-κB nuclear translocation, thereby suppressing downstream pro-inflammatory cytokine expression. (F-G) Real-time quantitative PCR results revealed that Aβ1–42 oligomers induced 6.06 ± 0.25-fold and 9.88 ± 0.25-fold increases in IL-1β and TNF-α mRNA expression, respectively (P < 0.001). C-K treatment downregulated these levels to 51.65 ± 8.46 % and 56.12 ± 2.64 % of the model group (*P < 0.001). Note: Data are presented as mean ± SD (n = 3). *P < 0.05, **P < 0.01, ***P < 0.001. C-K: Ginsenoside C-K.

The NF-κB signaling pathway, a core molecular mechanism regulating inflammatory responses, plays a pivotal role in neurodegenerative disease progression. To investigate the regulatory mechanism of GCK on Aβ1–42 oligomer-induced neuroinflammation, an in vitro inflammatory model was established by stimulating murine microglial BV2 cells with Aβ1–42 oligomers at a pathological concentration (32 nM). Western blot analysis revealed significant upregulation of phosphorylated IκBα (p-IκBα, Ser32) and nuclear phosphorylated NF-κB p65 (p-NF-κB p65, Ser536) protein expression in the model group, indicating specific activation of the NF-κB signaling pathway. Further quantitative real-time PCR and ELISA assays demonstrated that mRNA levels of pro-inflammatory cytokines IL-1β and TNF-α increased by 6.06 ± 0.25-fold and 9.88 ± 0.25-fold (P < 0.001), respectively, while their secreted protein concentrations rose to 18.31 ± 1.34 pg/mL and 23.08 ± 0.40 pg/mL (P < 0.01). Notably, pretreatment with GCK (4 μM) exhibited dose-dependent inhibitory effects on these inflammatory markers. At the molecular level, GCK treatment significantly suppressed IκBα phosphorylation and blocked NF-κB p65 nuclear translocation. Concurrently, IL-1β and TNF-α gene expression decreased to 51.65 ± 8.46 % and 56.12 ± 2.64 % of model group levels (P < 0.001), with corresponding reductions in protein secretion (P < 0.01). These results demonstrate that GCK effectively alleviates Aβ1–42 oligomer-induced neuroinflammation by inhibiting aberrant activation of the IκBα/NF-κB signaling pathway (Fig. 3).

### 3.4 GCK counteracts Aβ1–42 oligomer-induced neurotoxicity through direct and indirect mechanisms

**Fig. 4:**
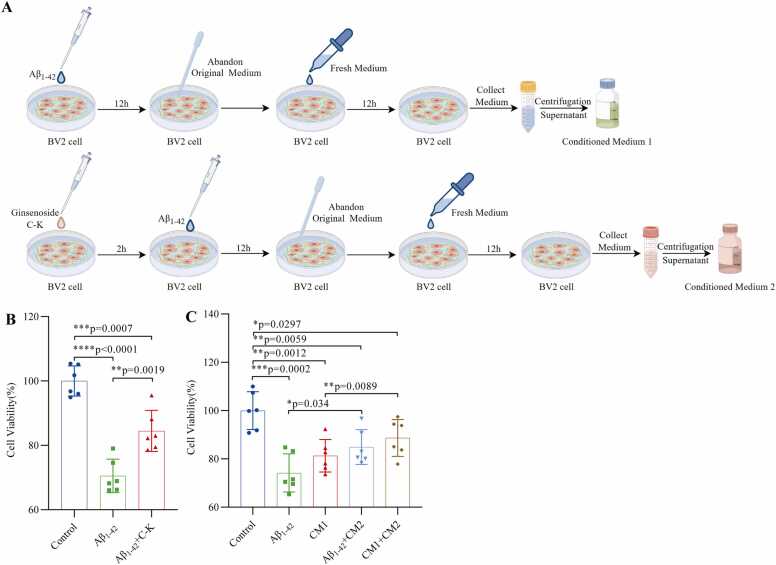
GCK counteracts Aβ1–42 oligomer-induced neurotoxicity through direct and indirect mechanisms. (A) Schematic workflow for conditioned medium preparation. Top panel: BV2 cells were treated with Aβ1–42 oligomers (32 nM) for 12 h, followed by medium replacement and additional 12 h incubation. The supernatant was collected and centrifuged to prepare Conditioned Medium 1 (CM1). Bottom panel: BV2 cells were pretreated with GCK (4 μM) for 2 h before Aβ1–42 oligomer treatment. Conditioned Medium 2 (CM2) was prepared using the same protocol. (B) Evaluation of GCK's direct protective effects. Compared to the control group, Aβ1–42 oligomer treatment significantly reduced HT22 cell viability (70.51 ± 4.75 %, ***p < 0.0001). GCK pretreatment markedly improved cell viability (84.49 ± 5.82 %, **p < 0.01 vs. Aβ group). (C) Evaluation of GCK's indirect protective effects. Both Aβ1–42 oligomers and CM1 treatment significantly decreased HT22 cell viability compared to controls (77.34 ± 5.21 % and 81.28 ± 6.14 %, respectively; **p < 0.01). In contrast, CM2 treatment significantly enhanced cell viability (88.69 ± 6.97 %, **p < 0.01 vs. CM1 group). Note: Data are expressed as mean ± standard deviation (n = 6). *p < 0.05, **p < 0.01, ***p < 0.001. Abbreviations: C-K, Ginsenoside Compound K; CM1, Conditioned Medium 1; CM2, Conditioned Medium 2.

To elucidate the neuroprotective mechanisms of GCK (Ginsenoside C-K), this study investigated its effects at both direct and indirect levels. First, we validated GCK's antagonistic effect against direct neurotoxicity induced by Aβ1–42 oligomers. Results showed that after 24-h treatment with Aβ1–42 oligomers (32 nM), HT22 cell viability significantly decreased to 70.51 ± 4.75 % (vs. control group, p < 0.0001). However, pretreatment with GCK (4 μM) for 2 h notably restored cell viability to 84.49 ± 5.82 % (vs. Aβ group, p < 0.01), demonstrating GCK's direct neuroprotective effect (Fig. 4B).

Second, to explore whether GCK indirectly protects neurons by modulating the neuroinflammatory microenvironment, we established a conditioned medium model (Fig. 4A). Specifically, Conditioned Medium 1 (CM1) was prepared by collecting supernatant from BV2 cells treated with Aβ1–42 oligomers for 24 h (12 h + 12 h). Conditioned Medium 2 (CM2) was obtained by pretreating BV2 cells with GCK for 2 h before identical Aβ1–42 oligomer treatment. The study revealed that CM1 treatment reduced HT22 cell viability to 81.28 ± 6.14 % (p < 0.01), similar to the Aβ1–42 oligomer-treated group, suggesting that activated microglia may exacerbate neuronal damage by releasing pro-inflammatory factors. In contrast, CM2 treatment significantly increased HT22 cell viability to 88.69 ± 6.97 % (vs. CM1 group, p < 0.01), indicating that GCK pretreatment may promote microglial polarization toward an anti-inflammatory phenotype (Fig. 4C).

In summary, this study confirms GCK's dual neuroprotective mechanisms: ① alleviates Aβ-induced synaptic damage in neurons by inhibiting NF-κB nuclear translocation and downregulating the secretion of IL-1β and TNF-α; and ② remodeling of the neuroinflammatory microenvironment by regulating microglial polarization. These findings provide new insights into GCK's mechanistic actions and experimental evidence for developing multi-target therapeutic strategies against Alzheimer's disease.

## 4 Discussion

Alzheimer's disease (AD), a classic multifactorial neurodegenerative disorder, is characterized by hallmark pathological features including Aβ aberrant deposition forming senile plaques, hyperphosphorylated tau protein-induced neurofibrillary tangles, and synaptic plasticity impairment-triggered neural network dysfunction as core pathological mechanisms (Basurto-Islas et al., 2025). Although current clinical therapeutic strategies encompass cholinesterase system modulators (e.g., donepezil), glutamate receptor antagonists (e.g., memantine), and emerging immune-targeted therapies (e.g., anti-Aβ monoclonal antibodies) (Conti et al., 2023; Haddad et al., 2022; Ippoliti et al., 2023), existing treatments still demonstrate significant limitations in disease modification, failing to achieve stable disease progression control or pathological reversal. Existing experimental data primarily rely on HT22 cells, whereas models using differentiated neurons may provide a more accurate representation of pathological conditions and responses. In this context, natural compounds with multi-target regulatory properties and biosafety have become a crucial research direction for neurodegenerative disease therapeutic development (Bhattacharya et al., 2025). Building on pharmacological research foundations, this study investigates the neuroprotective effects of GCK through multiple pathways in an in vitro AD model, while comprehensively exploring its multidimensional neuroprotective mechanisms and translational medical value.

### 4.1 Multidimensional evidence supports the neuroprotective effect of GCK

This study evaluated the role of GCK in Aβ1–42 oligomer-induced neurotoxicity and inflammatory damage through multiple indicators including proliferative activity, migratory capacity, and apoptosis rate. Experiments on HT22 and BV2 cells revealed: Within safe concentration ranges (HT22: ≤6 μM; BV2: ≤8 μM), GCK exhibited only mild, reversible cytotoxicity. After treatment with 32 nM Aβ1–42 oligomers, both HT22 and BV2 cells showed significantly reduced viability alongside pronounced inflammatory responses and apoptotic tendencies. Administration of 4 μM GCK effectively mitigated Aβ1–42 oligomer-induced neural damage, restoring HT22 cell viability to over 92 % of baseline levels and markedly improving abnormal pro-inflammatory factor secretion and migration rates in microglia (BV2 cells).

These findings align closely with the emerging perspective of "multi-pathway synergistic inhibition of Aβ toxicity and neuroinflammation." Previous studies demonstrate that ginsenosides exert composite regulatory effects on synaptic plasticity, mitochondrial energy homeostasis, and microglial activation, with their pleiotropic and targeted properties garnering significant attention in anti-AD research (Jiang et al., 2024; Nguyen et al., 2024; Zhang et al., 2025). This study provides new evidence for their specific anti-AD mechanisms and further solidifies the feasibility of GCK as a potential neuroprotective agent.

### 4.2 Potential molecular mechanisms and signaling pathway networks underlying the anti-Aβ toxicity

#### 4.2.1 Aβ42 and microglial activation with inflammatory pathways

Aβ42, the most toxic and aggregation-prone Aβ subtype in Alzheimer’s disease (AD) progression, binds to microglial surface receptors, triggering cascading inflammatory responses. These include NF-κB signaling cascade activation and massive release of pro-inflammatory cytokines (IL-1β, TNF-α, IL-6) (Haessler et al., 2024). Microglia act as a "double-edged sword" in AD pathology: while they clear aggregated Aβ, excessive stimulation leads to inflammatory factor secretion and neuronal damage (Bui et al., 2024). This study found that 32 nM Aβ42 significantly reduced BV2 cell viability and induced severe inflammation, whereas pretreatment with 4 μM GCK restored BV2 cell activity from 78.58 ± 4.37 % to near-normal levels and markedly reduced apoptosis. This suggests GCK may directly or indirectly suppress microglial overactivation; further studies in differentiated neuronal models will be essential to confirm the applicability of these results in vivo. Western blot data further revealed that GCK downregulated NF-κB p65 phosphorylation and upregulated IκBα expression. Upstream signaling pathways likely involve the TLR4/MyD88 or MAPK/NF-κB axis (Chen et al., 2025; Lv et al., 2024).

#### 4.2.2 GCK’s impact on mitochondrial function and energy homeostasis

Aβ42 oligomers impair mitochondrial function in neurons and microglia, disrupting ATP synthesis and causing abnormal reactive oxygen species (ROS) accumulation, thereby activating apoptotic pathways (Zhang et al., 2025). Using Annexin V-FITC/PI dual-staining flow cytometry, this study observed a significant increase in apoptosis in Aβ42-treated groups, while GCK reduced apoptosis rates by 52 % and improved mitochondrial membrane potential recovery. Combined with prior literature, GCK may exert anti-apoptotic effects by modulating mitochondrial membrane potential (ΔΨm) and regulating the Bcl-2/Bax ratio (Li et al., 2012). Additionally, mitochondria-ER contact sites (MERCs), critical for calcium homeostasis and protein folding balance (Barrera et al., 2021), could be further analyzed via transmission electron microscopy and calcium tracing to systematically validate GCK’s regulatory role in ER stress-mitochondrial injury pathways (Piao et al., 2019).

#### 4.2.3 Potential antioxidant stress and autophagy modulation effects

Beyond inflammation and mitochondrial dysfunction, Aβ42 accumulation-driven oxidative stress and autophagy impairment are key pathological mechanisms in AD. Studies indicate GCK not only scavenges free radicals but also activates the Nrf2/HO-1 pathway to enhance antioxidant and detoxification capacity (Li et al., 2024). A recent Molecular Psychiatry study highlighted that Aβ-phosphorylated tau interactions exacerbate autophagic-lysosomal pathway burdens in neurons, creating a vicious cycle (Ju et al., 2024). Although this study did not focus on autophagy-related proteins (LC3, p62) or lysosomal function, combining Western blot and immunofluorescence assays could clarify how GCK influences autophagic flux and exerts synergistic protection across multiple pathological networks.

### 4.3 Advantages over existing therapeutic approaches and clinical translation value

#### 4.3.1 Multi-target synergy and safety

Unlike single-target drugs (e.g., donepezil, which solely inhibits acetylcholinesterase, or anti-Aβ monoclonal antibodies that primarily clear amyloid plaques), GCK exerts therapeutic effects through multiple pathways, including inflammatory pathways, mitochondrial regulation, anti-apoptosis, and potential autophagy modulation (Fu et al., 2023; Kim et al., 2022; Mao et al., 2024; Yan et al., 2024a, Yan et al., 2024b). Safety evaluations at the cellular level, such as CCK-8 assays in HT22 and BV2 cells, demonstrated no significant toxicity at GCK concentrations below 6 μM/8 μM, well under conventional cytotoxicity thresholds. This aligns with findings by Wan et al., where ginsenoside compound K inhibited nuclear factor-κB by targeting annexin A2 (Wang et al., 2019a, Wang et al., 2019b). These results confirm that GCK, within appropriate dosage ranges, exhibits a favorable safety profile and multi-target regulatory efficacy; however, future studies should focus on differentiated neuronal models to assess the full therapeutic potential and safety in a more physiologically relevant context. However, all safety data in this study were obtained from cell models and cannot be used as a basis for predicting in vivo safety.

### 4.4 Preclinical research and formulation development

As shown in this study and prior literature, GCK demonstrates advantages in improving blood-brain barrier stability and metabolic stability (Li et al., 2021; Yan et al., 2024a, Yan et al., 2024b). Pharmacokinetic analyses indicate that its bioavailability can be significantly enhanced using technologies such as nanostructured lipid carriers, microspheres, or phospholipid complexes (Singh et al., 2018). Future optimization of oral or injectable formulations, combined with systematic validation of spatial memory, cognitive function, and peripheral safety metrics in AD mouse models (e.g., APP/PS1, 5× FAD), could accelerate its translation to clinical applications.

#### 4.4.1 Potential for combination with other therapeutic strategies

Given the complexity of AD, which involves multiple concurrent pathways, single therapies have limited efficacy. The call for "multi-modal strategies" in AD treatment is growing—for example, combining anti-Aβ monoclonal antibodies with chronic inflammation modulators or pairing novel synaptoplastic molecules (e.g., BDNF analogs) with neuroprotective agents (Laczo et al., 2020; Yang et al., 2024). Due to its multi-target network effects, GCK is well-positioned to synergize with other anti-AD drugs in a "cocktail therapy" approach, addressing Aβ deposition, neuroinflammation, and synaptic dysfunction simultaneously. Combination therapies reduce overreliance on single molecular targets and maximize the strengths of individual agents for holistic treatment optimization. Thus, this study provides critical insights for designing future "GCK+ antibody therapy" or "GCK+ recombinant protein therapy" regimens.

### 4.5 Study limitations

This study primarily focused on HT22 and BV2 cell lines. While these models preliminarily simulate neuronal and microglial damage processes in AD pathology, they still differ from the in vivo environment, such as lacking multicellular interactions involving astrocytes, brain microvascular endothelial cells, and other cell types. The aggregation state, purity, and quality control of Aβ oligomers significantly impacted experimental outcomes; integrating proteomics and biophysical analyses could more precisely assess their true aggregation forms. Reliance solely on viability assays, migration experiments, and Western blotting failed to capture more dynamic molecular pathways. Further exploration of GCK’s functional network requires multi-omics approaches (e.g., transcriptomics, epigenomics, metabolomics) for deeper mechanistic insights.

## 5 Conclusion

In summary, this study preliminarily demonstrated in an in vitro model that GCK effectively inhibits Aβ1–42 oligomer-induced neurotoxicity and inflammatory responses, while exhibiting significant protective effects against microglial (BV2) activation and neuronal (HT22) apoptosis. The molecular mechanisms may be linked to the regulation of the NF-κB signaling pathway, suppression of apoptosis, and enhancement of cell proliferation functions. This research not only provides novel insights and experimental foundations for utilizing active components of traditional Chinese medicine in Alzheimer's disease (AD) intervention but also highlights the necessity of using differentiated neuronal models in future studies to enhance the translational relevance of our findings. With advancements in multi-omics and high-throughput screening technologies, a more comprehensive elucidation of the interplay among 'GCK-Aβ-neuroinflammation' and their roles in AD pathogenesis will become feasible, particularly when integrating studies using differentiated neuronal models. Further validation of GCK's efficacy and safety in higher-order models (such as animal studies and clinical trials) could potentially pioneer new technical pathways for urgently needed AD drug development in clinical practice, thereby advancing precision prevention and comprehensive treatment strategies for AD.

## CRediT authorship contribution statement

**Kefeng Zhang:** Supervision, Funding acquisition, Formal analysis. **Cunxin Zhang:** Validation, Software, Investigation, Formal analysis. **Chenghu Xie:** Writing – original draft, Investigation, Formal analysis, Data curation. **Shanshan Zhang:** Writing – review & editing, Supervision, Resources, Methodology, Funding acquisition, Conceptualization.

## Consent for publication

All co-authors have agreed to the submission of the final manuscript.

## Ethics approval

This study does not involve human medical ethics or animal ethics.

## Funding

Shandong Province Medical and Health Science and Technology Development Program Project (202403070438) and Key R&D Program of Jining (2024YXNS190, 2023YXNS147).

## Conflicts of Interest

The authors have no relevant financial or non-financial interests to disclose.
